# Outcomes of Bridging Therapy in Liver Transplantation for Hepatocellular Carcinoma

**DOI:** 10.3390/jcm13226633

**Published:** 2024-11-05

**Authors:** Piotr Remiszewski, Paweł Topolewski, Dariusz Łaski, Anna Drobińska

**Affiliations:** Department of General, Endocrine and Transplant Surgery, Medical University of Gdańsk, 80-210 Gdańsk, Poland; ptopolewski@gumed.edu.pl (P.T.); dariusz.laski@gumed.edu.pl (D.Ł.); annad@gumed.edu.pl (A.D.)

**Keywords:** liver transplantation, hepatocellular carcinoma, bridging, transplant oncology

## Abstract

**Background**: Liver transplantation (LT) is a method for treating hepatocellular carcinoma (HCC) with satisfactory outcomes. One of the novel methods for predicting LT outcomes is the Metroticket 2.0 model. The disease in patients initially within the Milan criteria (MC) may progress while on a transplantation waitlist; thus, various transplantation bridging therapy (BT) methods are proposed for patients to stay within the MC and optimize the LT outcome. **Methods**: We performed a retrospective analysis of patients who underwent LT for HCC at an oncological and transplantation center in northern Poland. Patients who underwent (n = 10) or did not undergo (n = 11) BT were included. The primary endpoints of the study were mortality among the patients, HCC recurrence, and Metroticket 2.0 scores based on LT qualification results and explant pathology outcomes. The median follow-up length was 44.03 months. **Results**: Patients who underwent BT had significantly lower Metroticket 2.0 scores and greater AFP concentrations at baseline. At LT, there was no significant difference in Metroticket 2.0 scores or AFP concentrations between the groups. Explant Metroticket 2.0 scores were significantly lower in patients who received BT. A complete pathologic response was achieved in 30.0% of patients who underwent BT. The recurrence-free survival rates were 100% and 90.91% in patients who underwent and did not undergo BT, respectively. Overall survival was 80.0% and 81.81% in patients who underwent and did not undergo BT, respectively. **Conclusions**: BT should be considered only as a means of remaining within the LT criteria. Routine BT does not appear to be justified for LT patients.

## 1. Introduction

Hepatocellular carcinoma (HCC) represents up to 85% of all primary liver cancer cases and is an important health and health-related economic burden in the global healthcare system [1,2,3]. Liver transplantation (LT) is a method for treating HCC with satisfactory outcomes. The Milan criteria (MC) proposed by Mazzaferro et al. remain a benchmark with respect to qualifying patients with HCC for LT [4,5]. Patients within the MC have an optimal 5-year survival rate of at least 70% after LT [5]. The disease in patients initially within the MC may progress while on a transplantation waitlist; thus, various transplantation bridging therapy (BT) methods are proposed for patients to stay within the MC and optimize the LT outcome. Patients with HCC, initially within the MC, who receive bridging therapies have greater chances of favorable LT outcomes [6]. However, data regarding the survival benefit of bridging therapies in patients with HCC undergoing LT are still lacking. One of the methods for predicting LT outcome is the Metroticket 2.0 model, which is based on the level of alpha-fetoprotein (AFP), tumor size, and tumor number and allows the prediction of the risk of death from HCC-related factors after LT.

In this retrospective study, we aimed to evaluate the impact of bridging therapies on predicted and actual post-LT survival and the possibility of achieving a complete pathologic response in patients with initially lower Metroticket 2.0 scores than in patients with initially higher Metroticket 2.0 scores who did not undergo bridging therapy.

## 2. Materials and Methods

### 2.1. Study Design

This retrospective study included patients with HCC who did or did not undergo locoregional treatment (radiofrequency ablation (RFA) or/and transarterial chemoembolization (TACE)) prior to LT at the oncological and transplantation center in northern Poland between April 2018 and January 2023. During that period, a total of 158 LTs were performed. The patient, intervention, comparison, and outcome (PICO) questions are presented in Table 1. Patient variables and follow-up data were assessed from medical records. Outcomes of biochemical and radiological (contrast-enhanced computed tomography) examinations were assessed at the time of LT, on the day of LT, and at the latest follow-up examination. Standardized histopathological examinations were performed on the explanted livers. Every patient was discussed at a multidisciplinary board meeting and was qualified for locoregional therapy with the intention of receiving treatment if (a) locoregional therapy could be a radical HCC treatment (achieving total tumor necrosis) and other indications for LT were present (chronic liver failure with episodes of decompensation), (b) the expected time on the transplant waitlist was long (over 4 months), (c) the tumor was progressing in size, or (d) the tumor was border-sized in the MC (single tumor over 40 mm or more two or three tumors with one sizing at least 20 mm). The size and number of tumors were evaluated via standard criteria [7] in computed tomography with contrast medium or magnetic resonance imaging at baseline and after each treatment session. All patients receiving locoregional therapy were followed up with a radiological examination (up to three months in patients receiving TACE, and up to six weeks after RFA). If the radiological diagnosis of HCC was not conclusive, a liver biopsy was performed.

### 2.2. Study End Points

The primary endpoints of the study were mortality among the patients, HCC recurrence, Metroticket 2.0 scores based on LT qualification results and explant pathology, and changes in the AFP concentration.

### 2.3. Data Collection and Statistical Methods

Data on patient age, sex, primary liver disease, number and type of locoregional treatment, beginning of qualification for LT, LT date, number and size of lesions in the first radiological examination at LT qualification, biochemical examination results (AFP, creatinine, bilirubin, INR, sodium and albumin concentrations), explant histopathology examination (number, size and grade of the tumors, extranodular infiltration, and vascular invasion), and clinical data (presence of ascites, dialysis, and encephalopathy) were obtained from the internal medical history system. The Metroticket 2.0, MELD, and Child-Pugh (CP) scores were calculated based on medical history data. The Metroticket 2.0 score was calculated via a calculator at http://www.hcc-olt-metroticket.org/ (accessed on 1 November 2024) by Vicenzo Mazzaferro et al. [8].

Overall survival (OS) and recurrence-free survival (RFS) were estimated by Kaplan-Meier analysis. The normality of the data was checked using the Shapiro-Wilk normality test. For unpaired data with no normal distribution, the Mann-Whitney test was used; for paired data with no normal distribution, the Wilcoxon test was used; and for paired data with a normal distribution, the paired *t* test was used. *p* values < 0.05 were considered significant. Analyses were performed using Prism Version 9.4.1 (GraphPad 1994–2022, GraphPad Software, Boston, MA, USA).

## 3. Results

During the study period, a total of 158 LTs were performed. The analysis identified 21 patients who underwent deceased donor orthotopic LT due to HCC, initially within the MC. The HCC diagnosis was based on radiological and histopathological examinations. Fifteen patients also had chronic liver failure manifesting as a bleeding from esophageal varices in five patients, refractory ascites in three patients, hepatic encephalopathy in two patients, and at least two mentioned symptoms in five patients. There were no patients who could not receive locoregional therapy because of poor liver function. There were no patients who were considered as an LT candidate and delisted after receiving locoregional therapy. There were no patients who were delisted due to tumor progression. There were no patients who were waitlisted and received or did not receive BT and were not yet transplanted. All patients underwent the same immunosuppressive protocol, which included perioperative methylprednisolone, steroids, and tacrolimus. There was no need for repeated courses of steroids for episodes of rejection in the cohort. The immunosuppressive protocol did not significantly differ among the patients in the perioperative or early post-transplant period.

The patients in the study cohort were allocated into two groups: group A (with locoregional treatment prior to LT, n = 10) and group B (without locoregional treatment prior to LT, n = 11). The reasons for performing bridging therapy were as follows: MC border tumor size in one patient, progression of tumor size in four patients, and both border tumor size and size progression in five patients. Eight patients received RFA, one patient received TACE, and one patient received both RFA and TACE. Four patients received singular RFA, three patients received two RFA sessions, and one patient received three RFA sessions. Patients who received TACE receive TACE in two sessions over a four-week period according to the standard protocol used in our center. The patient who received both RFA and TACE received one RFA session and two TACE sessions. The baseline characteristics of the study cohort and divided subgroups are presented in Table 2.

**Figure 1:**
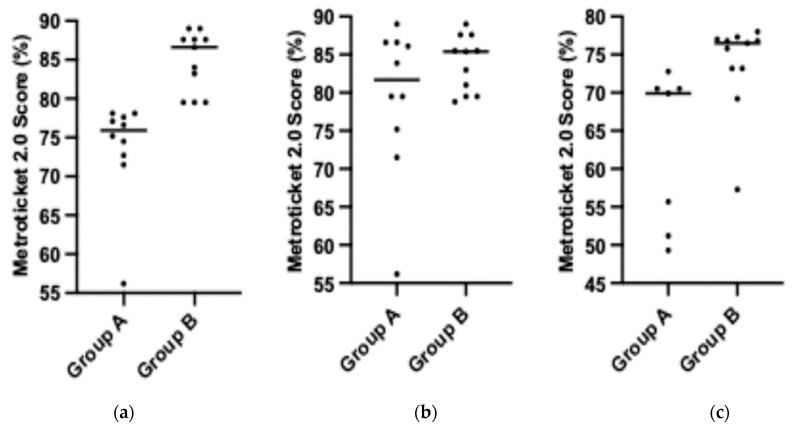
Metroticket 2.0 scores (%) in subgroups of the study cohort: (**a**) baseline scores (*p* < 0.0001); (**b**) liver transplantation scores (*p* = 0.4340); (**c**) explant scores (*p* = 0.0036).

**Figure 2:**
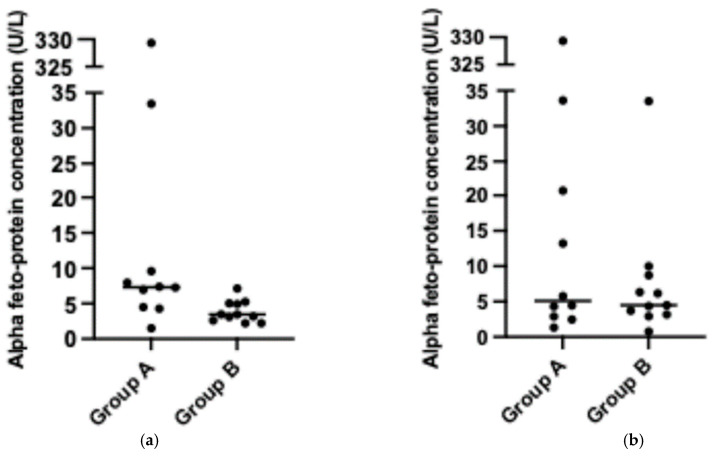
Alpha-fetoprotein concentration: (**a**) baseline (*p* = 0.012) and (**b**) at liver transplantation (*p* = 0.7564).

The Metroticket 2.0 score was significantly lower (*p* < 0.0001) in patients who received BT than in patients who did not receive BT (Figure 1, panel a). The AFP concentration was significantly greater in patients in group A (*p* = 0.012) (Figure 2, panel a). Patients who received BT had significantly greater tumor sizes (*p* = 0.0197). The other baseline characteristics did not differ among the groups.

The group characteristics at LT are presented in Table 3. The results of the waitlist radiological follow-up (according to the modified Response Evaluation Criteria In Solid Tumors [9]) were as follows: four patients had progressive disease, two patients had stable disease, one patient had a partial response, and three patients had a complete response. The AFP concentrations did not differ significantly among the groups or between the groups (baseline compared with LT). Metroticket 2.0, MELD, and CP scores and AFP concentrations were not significantly different between the groups (Figure 2, panel b). The in-group analysis revealed that the Metroticket 2.0 scores were significantly higher at transplant than at baseline in group A (Figure 1, panel b). The other results were not significantly different between the groups.

The outcomes of histopathological examination of the explanted livers are presented in Table 4.

In three livers of the patients in group A, total pathological response was confirmed. The Metroticket 2.0 score calculated based on the results of explant histopathological examination was greater in group B than in group A, and the difference was statistically significant (*p* = 0.0036) (Figure 1, panel c). An in-group analysis revealed that the explant size of the tumors was significantly greater in group B than at baseline (*p* = 0.002); however, no significant difference was found in group A. The Metroticket 2.0 score calculated based on the results of the explant examination, was not significantly different between the groups. Moreover, the in-group analysis revealed that explant Metroticket 2.0 scores were significantly higher than baseline scores in group A (*p* = 0.0117). The difference between the baseline and explant Metroticket 2.0 scores was not statistically significant (*p* = 0.1562). Six patients were found to be outside the MC based on explant pathology: five in group A and one in group B.

**Figure 3:**
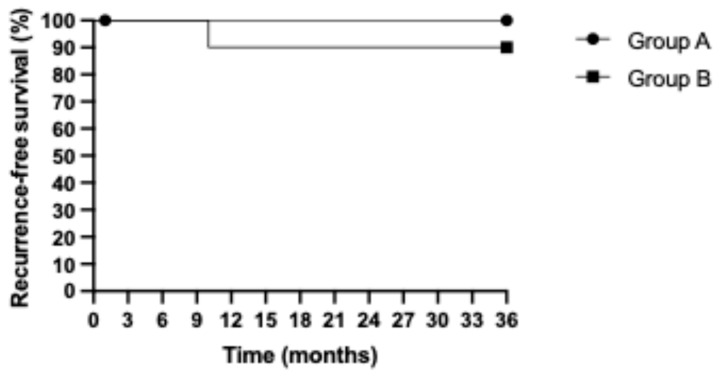
Three-year recurrence-free survival of patients in the two subgroups of the study cohort (*p* = 0.3173).

**Figure 4:**
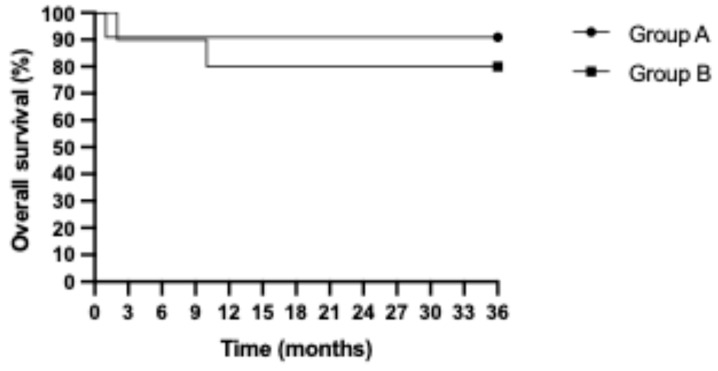
Three-year overall survival of patients in the two subgroups of the study cohort (*p* = 0.5249).

The median follow-up length was 44.03 months. During the follow-up period, four patients died (two in each group). The details of the four patients who died are presented in Appendix A. The median OS was 42.71 months (43.55 months and 41.46 months in groups A and B, respectively). The three-year RFS and OS Kaplan-Meier curves are presented in Figure 3 and Figure 4, respectively. No statistically significant differences in three-year RFS (*p* = 0.3173) or OS (*p* = 0.5249) were detected in the group analysis. The recurrence-free survival rates were 100% and 90.91% in groups A and B, respectively. The overall survival was 80.0% and 81.81% in groups A and B, respectively.

## 4. Discussion

Bridging therapies aim to decrease dropout rates from transplantation waitlists and improve LT outcomes by decreasing recurrence-free survival. Locoregional therapies such as RFA and TACE can be used both as downstaging to LT and as BT to LT. Patients who undergo RFA or/and TACE have a chance of tumor destruction or stabilization, which can improve the outcome of LT. When complete tumor necrosis is achieved, patients may benefit from having completely inactivated oncological disease at the time of LT and, therefore, a better chance of favorable LT outcomes. Currently, patients with border-sized tumors, aggressive tumor biology, or possibly longer waiting times undergo locoregional BT. Direct data from prospective studies regarding the superiority of any bridging method are lacking, and comparative prospective studies may be biased in terms of patient allocation [10]. Most of the data reported include TACE as a method of bridging [10]. In our study, we included RFA as the main modality of bridging. TACE may cause vascular damage and potentially increase hepatectomy difficulty; however, more data on this topic are lacking.

In our study, the patients in group A had significantly lower Metroticket 2.0 scores when qualified for LT and BT. None of the patients in either group who qualified for LT due to HCC dropped out of the waitlist. Locoregional therapy resulted in a significant change in the AFP concentration and, therefore, favorable possible outcomes on the day of LT. The effect of BT was apparent, as the AFP concentration, a mass-dependent marker of the presence of HCC, decreased due to the use of BT. Thus, we can assume that the disease became less active, which was confirmed by the outcomes of standardized histopathological examination. The explant Metroticket 2.0 score was significantly lower in group A and the OS was similar in both groups. There were no cases of recurrence in group A.

In three (30.0%) patients, total inactivation of HCC was achieved, and the median degree of necrosis of the tumors in the explanted livers was 90%. The 70.0% of tumors consisting of viable tumor tissue can be explained by the more aggressive tumor biology in this group and faster recurrences or inadequate locoregional treatment. Most of the explanted tumors consisted of necrotic tissue, and the amount of actual active cancer tissue was significantly lower than the size of the tumor reported on histopathological examination. Therefore, we can assume that the actual Metroticket 2.0 scores based on the explant pathology are lower than those included in the analysis. However, methods to provide statistical proof of such data interpretation are lacking. All patients in whom total necrosis of the tumor was achieved received RFA only.

Large multicenter cohort studies have shown that locoregional therapies such as RFA and TACE or RFA combined with TACE improve overall and recurrence-free survival after LT [11]. It has also been shown that complete tumor necrosis is correlated with LT outcomes in terms of post-transplant recurrence and survival [12]. On the other hand, some researchers have suggested that bridging therapies and a complete pathologic response are not associated with favorable long-term outcomes after LT for HCC patients [13,14,15,16,17]. Xu et al. suggested that neoadjuvant-therapy-induced necrosis is associated with increased lymphangiogenesis and, therefore, an increased risk of post-transplantation lymphatic metastases [18]. Our results are consistent with those of a previous study by Ramanathan R. et al. [19]; however, we believe that because of new data in the field, there is an ambiguity yet to be resolved. The main inconsistency in the literature is whether BT improves LT outcomes, as the published results disagree [20]. We believe that our research is a valuable contribution to the discussion, as we prove that bridging therapy does not contribute to better survival after LT and provide additional evidence supporting the use of bridging therapy prior to LT as a means of disease stabilization. Lamarque et al. suggested that performing LT bridging and delaying LT by increasing the waitlist time while bridging does not negatively impact LT outcomes [21]. This approach has the potential to allow for the redistribution of organs to patients in more urgent need of LT. Butcher et al. suggested that neoadjuvant TACE can be used for patients with longer expected waiting list times, as patients treated with TACE had LT outcomes similar to those of non-TACE patients; however, TACE patients had worse prognostic features than non-TACE patients did [22].

The limitations of this study include its retrospective nature, small number of patients, and two factors contributing to bias at one of the study endpoints, as already mentioned. In this case-series analysis, the small number of patients and bridging selection criteria are two sources of possible bias of our results; however, with opposing results, in our opinion, more data are needed on this subject. Every patient was discussed in a multidisciplinary board meeting, where selection for bridging was made. Apart from the small series, the results of our analysis have also been suggested previously. Additionally, the Metroticket 2.0 score is dependent on the AFP level; therefore, these two endpoint outcomes are correlated. The greatest bias of this study is in the relatively short waitlist time, which is much shorter in most other centers.

The indications are still unclear. Current guidelines tend to focus on whether to perform a BT, which BT technique to use, and whether the benefit of BT is visible in post-LT survival. Current evidence shows that BT is encouraged to prevent the LT delisting and may be associated with a transient increase in one-year post-LT survival in patients who underwent BT [6,20,23,24]. Recently, the delayed LT strategy in HCC patients receiving BT was investigated by Lamarque et al. [21]. The authors showed that the delayed strategy involving BT did not negatively impact LT outcomes, and had the potential to allow the redistribution of organs for patients in need of urgent LT. This research is an important argument for using BT for other reasons than favorable LT outcomes. If BT therapy can prolong the LT waitlist time, then the number of patients who can benefit from receiving an LT is greater and the overall results of HCC treatment improve, as LT is one of the best definitive HCC treatment methods. BT is encouraged when the expected LT waitlist time exceeds six months [25,26,27]; however, the tumor size and risk of progression was not addressed. In our small case series, the risk of progression over MC was one of the main indications for performing a BT, even if the expected LT waitlist time did not exceed six months. The risk of tumor progression over MC is rather based on clinical expertise and individual patient’s background. HCC is a heterogeneous tumor group with heterogeneous tumor biology and growth times [28]. Therefore, we support the statement that selecting the optimal LT strategy (with or without BT) should not be conducted without including the patient’s individual context. Also, response to BT may become an important indicator of LT outcomes. If the effect of locoregional therapy is insufficient, the risk of recurrence of HCC might increase [13,29,30,31]. Xu et al. [18] reported that partially necrotic HCC induced by locoregional therapy was associated with an increased risk of lymphatic metastases. Such phenomena are one of several possible explanations behind the lack of benefit between the BT and no-BT groups. Recently, the role of molecular-based therapies was discussed alone or addition to locoregional therapies. Sorafenib, lenvatinib, and regorafenib, which are oral multi-kinase inhibitors, were investigated as possible therapies in BT [32,33,34,35,36]. The current evidence on using molecular-based and systemic therapies in LT BT were discussed widely in previous reports [27,37]; however, their roles should not be omitted in discussing the future of LT bridging. Ongoing clinical trials are examining the molecular-based and systemic therapies alone or as an addition to locoregional therapies such as LT bridging. Immune-checkpoint inhibitors in combination with tyrosine kinase inhibitors or VEGF inhibitors likely represent the most promising approach. However, the concerns remain regarding post-LT rejection after bridging with immune-checkpoint inhibitors. The selection of BT modality is yet to be discussed, and multimodal techniques with the use of systemic therapies are encouraged due to favorable results; however, in our opinion, the main clinical problem remains in selecting the candidates for LT BT.

We suggest that the Metroticket 2.0 score calculated from explant pathology may differ between patients who underwent locoregional therapy prior to LT and those who did not. Future analyses, including analyses of the ability of the rate of tumor necrosis to predict LT outcomes and survival after LT, should be performed. Owing to inconsistencies in the reported results of locoregional treatment outcomes such as LT bridging, we suggest that there is a need for further analysis [37]. We suggest that BT does not currently appear to impact post-transplant recurrence under certain conditions in Poland.

## 5. Conclusions

Although complete pathologic response and transient improvement in Metroticket 2.0 scores were achieved, BT did not appear to improve post-LT survival. The correlation between the percentage of tumor necrosis according to explant pathology and the LT outcome has yet to be determined; however, it does not appear to improve post-LT survival. The mechanisms of such phenomena are yet to be determined. Our study’s unique aspects and findings are the clinical re-evaluation of the usefulness of bridging therapy to LT and the need for clarification of the present scientific evidence regarding the use of locoregional therapy as a bridge to LT. Owing to new data, we suggest that BT does not currently appear to impact post-transplant recurrence under certain conditions in Poland. Routine BT does not appear to be justified for certain groups of LT patients.

## Figures and Tables

**Table 1:** PICO criteria used in the study.

PICO	Description
Patient	Patients undergoing bridging locoregional treatment before liver transplantation due to hepatocellular carcinoma within Milan criteria.
Intervention	Locoregional treatment (radiofrequency ablation or/and transarterial chemoembolization) for hepatocellular carcinoma as a bridge to liver transplantation.
Comparison	Patients within Milan criteria without bridging locoregional treatment prior to liver transplantation.
Outcome	Metroticket 2.0 score, percentage of tumor necrosis, overall survival, recurrence-free survival.

**Table 2:** Baseline characteristics of the study cohort and subgroups.

Patient Characteristics	Group A (n = 10)	Group B (n = 11)	*p* Value
Sex M/F No.	7/3	11/0	0.0902
Age, median (IQR ^1^), years	60.5 (53.5–64.0)	57 (49.5–61.0)	0.7316
Primary liver disease: HCV ^2^ cirrhosis/HBV ^3^ cirrhosis/Mixed viral cirrhosis/Alcoholic cirrhosis/Other	5/1/3/0/1	1/4/1/5/0	-
Metroticket 2.0 score (%), median (IQR)	75.9 (73.15–77.48)	86.6 (81.35–87.60)	<0.0001
MELD ^4^ score, median (IQR)	9.5 (8.0–11.75)	12 (10.0–17.50)	0.0665
Child-Pugh score, median (IQR)	5.5 (5.0–6.75)	9 (6.0–9.0)	0.0737
Charlson Comorbidity Index, median (IQR)	6 (5.25–6.0)	7 (5.0–7.5)	0.7125
AFP ^5^ concentration (U/L), median (IQR)	7.33 (5.11–9.18)	3.46 (2.86–5.0)	0.012
Transplantation waiting time (days), median (IQR)	115.5 (41.75–139.75)	116 (41.0–300.0)	0.4679
Primary number of tumors, median (IQR)	1 (1–2)	1 (0–1)	0.0708
Primary size of tumors (mm), median (IQR)	17.5 (11.75–27.5)	8 (0–16.0)	0.0197

^1^ interquartile range. ^2^ Hepatitis C virus. ^3^ Hepatitis B virus. ^4^ Model for End-stage Liver Disease. ^5^ alpha-fetoprotein.

**Table 3:** Characteristics of the study cohort and subgroup comparisons at transplant.

Outcome	Group A (n = 10)	Group B (n = 11)	*p* Value
At-transplant Metroticket 2.0 score (%), median (IQR ^1^)	81.7 (76.28–86.48)	85.4 (80.25–86.55)	0.4340
At-transplant MELD ^2^ score, median (IQR)	9.5 (8.25–11.75)	10 (9.5–13.0)	0.3367
At-transplant Child-Pugh Score, median (IQR)	5.5 (5.0–7.0)	6 (5.0–9.0)	0.4414
At-transplant AFP ^3^ concentration (U/L), median (IQR)	5.10 (3.26–18.81)	4.48 (3.45–7.53)	0.7564

^1^ interquartile range. ^2^ Model for End-stage Liver Disease. ^3^ alpha-fetoprotein.

**Table 4:** Outcomes of histopathological examination of the explanted livers and explant Metroticket 2.0 scores.

Outcome	Group A (n = 10)	Group B (n = 11)	*p* Value
Explant Metroticket 2.0 score (%), median (IQR ^1^)	69.9 (53.5–70.5)	76.5 (73.2–76.9)	0.0036
Explant number of tumors, median (IQR)	1.5 (0.25–2)	1 (1.0–2.5)	0.6415
Explant size of tumors (mm), median (IQR)	31 (7–40)	20 (16–25)	0.2430
Explant tumor grade: I/II/III/IV	1/6/0/0	2/7/2/0	-
Microvascular invasion: yes/no	3/4	1/10	0.2451
Number of total necrotic tumors (%)	3 (30.0%)	-	-
Necrosis percentage in tumors (%), median (IQR)	90 (42.5–100)	-	-
Milan criteria: within/outside	5/5	10/1	0.0635

^1^ Interquartile range.

## Data Availability

The data presented in this study are available upon request from the corresponding author due to privacy concerns.

## Appendix A

We present the details of four patients who died during the follow-up.

### Appendix A.1. Patient 1, Group A

A 57-year-old female qualified for LT because of hepatitis C virus (HCV) liver cirrhosis, esophageal varices with a history of bleeding, and HCC within the MC (one lesion 8 mm in size). CP 7, CCI 6, MELD 14, at-transplant Metroticket 2.0 79.5%. A complete pathological response was found in the explanted liver. During the post-transplant course, primary graft nonfunction was suspected; however, liver function improved. Radiological diagnosis was performed, and no disturbance in hepatic blood flow was found. Eleven days after LT, the patient experienced cardiac arrest and died despite advanced life support. On autopsy, hepatic hypoxic necrosis and spread foci of necrosis in the brain were found to be direct causes of death.

### Appendix A.2. Patient 2, Group A

A 69-year-old female who qualified for LT because of HCV-related liver cirrhosis and HCC within the MC (two lesions larger than 15 mm) was included. CP 5, CCI 9, MELD 8, at-transplant Metroticket 2.0 76.6%. In the explanted liver, two HCC lesions were found, with a larger size of 45 mm (outside the MC). During the post-transplant course, the patient developed transplanted liver cirrhosis due to ischemic-type biliary lesions. She was admitted 43 months after LT due to liver jaundice and severe encephalopathy. Despite aggressive treatment, shortly after admission, the patient developed multiorgan failure and cardiac arrest and died. Multiple organ failure and transplanted liver failure were found to be direct causes of death.

### Appendix A.3. Patient 1, Group B

A 65-year-old male qualified for LT because of alcoholic liver cirrhosis, refractory ascites, and HCC within MC (one lesion with a size of 20 mm). CP 9, CCI 9, MELD 10, at-transplant Metroticket 2.0 85.4%. In the explanted liver, one HCC lesion with a size of 24 mm was found. Eleven months after LT, the patient was admitted because of fatigue and abdominal pain. Radiologically, hepatic abscesses and massive thoracic and abdominal lymphadenopathy were found. A biopsy was performed, and histopathological examination revealed the presence of poorly differentiated adenocarcinoma. During hospitalization, the patient’s condition worsened, and the patient died 305 days after LT. Multiorgan failure due to generalization of the oncological disease was found to be a direct cause of death.

### Appendix A.4. Patient 2, Group B

*A* 60-year-old male qualified for LT because of HBV liver cirrhosis and HCC within the MC (three lesions with the largest size of 21 mm). CP 5, CCI 7, MELD 10 at-transplant Metroticket 2.0 85.5%. In the explanted liver, two HCC lesions were found with sizes of 26 mm and 16 mm. In the second month after LT, the patient was admitted because of jaundice and fatigue. A diagnosis was made, and severe autoimmune hemolytic anemia was found. Despite aggressive treatment, the patient developed multiorgan failure and died 68 days after LT. Multiorgan failure caused by autoimmune hemolytic anemia was found to be a direct cause of death.
